# Timely diagnosis and treatment of sleep apnea reduce cardiovascular sequelae in patients with myocardial infarction

**DOI:** 10.1371/journal.pone.0201493

**Published:** 2018-07-30

**Authors:** Ming-Tzer Lin, Chao-Lun Lai, Pei-Lin Lee, Min-Huei Shen, Chong-Jen Yu, Chi-Tai Fang, Chi-Ling Chen

**Affiliations:** 1 Department of Internal Medicine, Hsiao Chung-Cheng Hospital, New Taipei, Taiwan; 2 Institute of Epidemiology and Preventive Medicine, College of Public Health, National Taiwan University, Taipei, Taiwan; 3 Center of Sleep Disorder, National Taiwan University Hospital, Taipei, Taiwan; 4 Department of Internal Medicine, National Taiwan University Hospital Hsin-Chu Branch, Hsin-Chu, Taiwan; 5 Center for Critical Care Medicine, National Taiwan University Hospital Hsin-Chu Branch, Hsin-Chu, Taiwan; 6 Departments of Internal Medicine, National Taiwan University Hospital, Taipei, Taiwan; 7 Center for electronics technology integration, National Taiwan University, Taipei, Taiwan; 8 Graduate Institute of Clinical Medicine, College of Medicine, National Taiwan University, Taipei, Taiwan; National Yang-Ming University, TAIWAN

## Abstract

**Background:**

The present study aimed to test if the temporal sequence between sleep apnea (SA) diagnosis and incident myocardial infarction (MI) was associated with the long-term mortality and cardiovascular event in a community-based cohort.

**Methods:**

We retrieved data from 9,453 incident MI patients between Jan. 1^st^ 2000 and Dec. 31^st^ 2012 from the Taiwan National Health Insurance Research Database. The study subjects included 207 MI patients with SA (SA-MI), further stratified into 110 with pre-existing SA before MI (SA-bMI) and 96 diagnosed with SA after MI (SA-pMI). The median follow-up period was 4.2 years. Propensity-score-matched controls were selected from 9,246 non-SA MI patients (non-SA-MI). The association of SA and outcomes including all-cause mortality and major adverse cardiac and cerebrovascular events (MACCEs) were analyzed by a Cox proportional hazards model.

**Results:**

The result showed that SA was not associated with mortality regardless of the timing of SA diagnosis. SA-pMI was associated with increased risk of MACCEs (Hazard ratio [HR]: 1.412, 95% confidence interval [CI]: 1.037~1.923, p = 0.029) including re-MI or revascularization and ischemic heart disease hospitalization. Such an association was most significant for SA diagnosed within one year after MI (HR: 2.029, 95% CI: 1.265~3.254, p = 0.003), which was not seen in patients treated with continuous positive airway pressure (CPAP).

**Conclusion:**

The temporal sequence and the time interval between SA diagnosis and incident MI was associated with the cardiovascular events after MI, especially within one year after MI. Early assessment for the presence of SA after incident MI and early CPAP intervention may reduce the risk of further adverse cardiovascular events.

## Introduction

Sleep apnea (SA) is characterised by the repeated collapse of upper airways during sleep, causing chronic intermittent hypoxia (CIH) and sleep fragmentation.[1] The prevalence rate of SA was about 30% in verified coronary artery disease (CAD) and up to 65.7% in acute myocardial infarction (MI).[2, 3] Obstructive apnea is more common than central apnea in MI patients and it does not resolve over time.[4, 5] The continuous positive airway pressure (CPAP) is the first-line treatment for SA which can improve daytime alertness, functional status, blood pressure, and quality of life in patients with daytime sleepiness. [6]

The association between SA and cardiovascular outcome has been investigated in many longitudinal cohorts but the findings are inconsistent. Shah et al. reported that, for patients without pre-existing CAD, SA was associated with a 1.06-fold increase in the risk of major cardiac events in a sleep-clinic cohort during an average 2.9-year follow-up.[7] Gottlieb et al. showed that the risk of coronary heart disease in SA patients only slightly increased in men ≤70 y/o (hazard ratio 1.1), but not in older men or in women in the community cohort, during the median follow-up of 8.7 years.[8] Lee et al. showed in a multi-center study that, for patients with CAD underwent successful percutaneous coronary intervention (PCI), SA was associated with a 57% increase in the risk of major adverse cardiac and cerebrovascular events (MACCEs) during the median follow-up of 1.9 years.[9] Yumino et al. found that, amongst patients with acute coronary syndrome with successful PCI, patients with SA had an 11.61-fold higher risk of MACCEs during the mean follow-up of 227 days in a single-center cohort.[10] These results show that the association between SA and cardiovascular outcome was highly influenced by patient source, pre-existing CAD, and duration of follow-up.

Possible mechanisms of how SA contribute to coronary events include CIH-induced oxidative stress and the subsequent inflammation, endothelial dysfunction, and platelet activation, eventually resulting in atheroma rupture and vasculothrombosis.[11, 12] Moreover, sympathetic hyperactivity could further increase oxygen consumption by increasing myocardial workload, further compromise the coronary perfusion which is already insufficient.[13] On the other hand, it has been proposed that SA associated CIH may induce ischemia preconditioning.[14] Evidence supporting the cardioprotective effect of SA included that MI patients with SA show higher circulating vascular endothelial growth factor (VEGF) and endothelial progenitor cells, better coronary collateral development, and lower troponin-T level compared to those without SA.[15–17]

Although SA has been associated with coronary events and stroke after MI, it has never been tested whether the temporal sequence or the time interval between SA diagnosis and incident MI was associated with the long-term mortality and cardiovascular event in a community-based cohort.[2] Previous studies excluded patients with hemodynamic instability, under oxygen supplementation, and new-onset heart failure symptom which may result in the exclusion of patients with a higher incidence of MACCEs. This study aimed to clarify the temporal association between SA diagnosis, all-cause mortality and MACCEs after incident MI in a large, population-based cohort with long-term follow-up to be free of selection bias. The association was analyzed with the stratification of the temporal sequence and the time interval between SA diagnosis and MI.

## Materials and methods

### Data sources

This study used data from the Longitudinal Health Insurance Database 2000 (LHID2000), which is a subset of the National Health Insurance Research Database (NHIRD) in Taiwan. The National Health Insurance program in Taiwan is a compulsory health insurance program which covers approximately 99.8% of the population of Taiwan in 2012 (http://nhird.nhri.org.tw/en/index.html). LHID2000 contains the claim data of one million beneficiaries that were randomly selected in 2000. The systematic sampling method allows no statistical difference in age, sex, and health care costs between the sample group and all enrollees. Personal privacy was protected by applying a consistent encryption procedure which maintains the linkage of all claims belonging to the same patient in the LHID2000 dataset. The information of this database such as prescription, diagnoses, and hospitalizations has been shown to be of high quality in previous studies.[18]

### Study population

**Fig 1:**
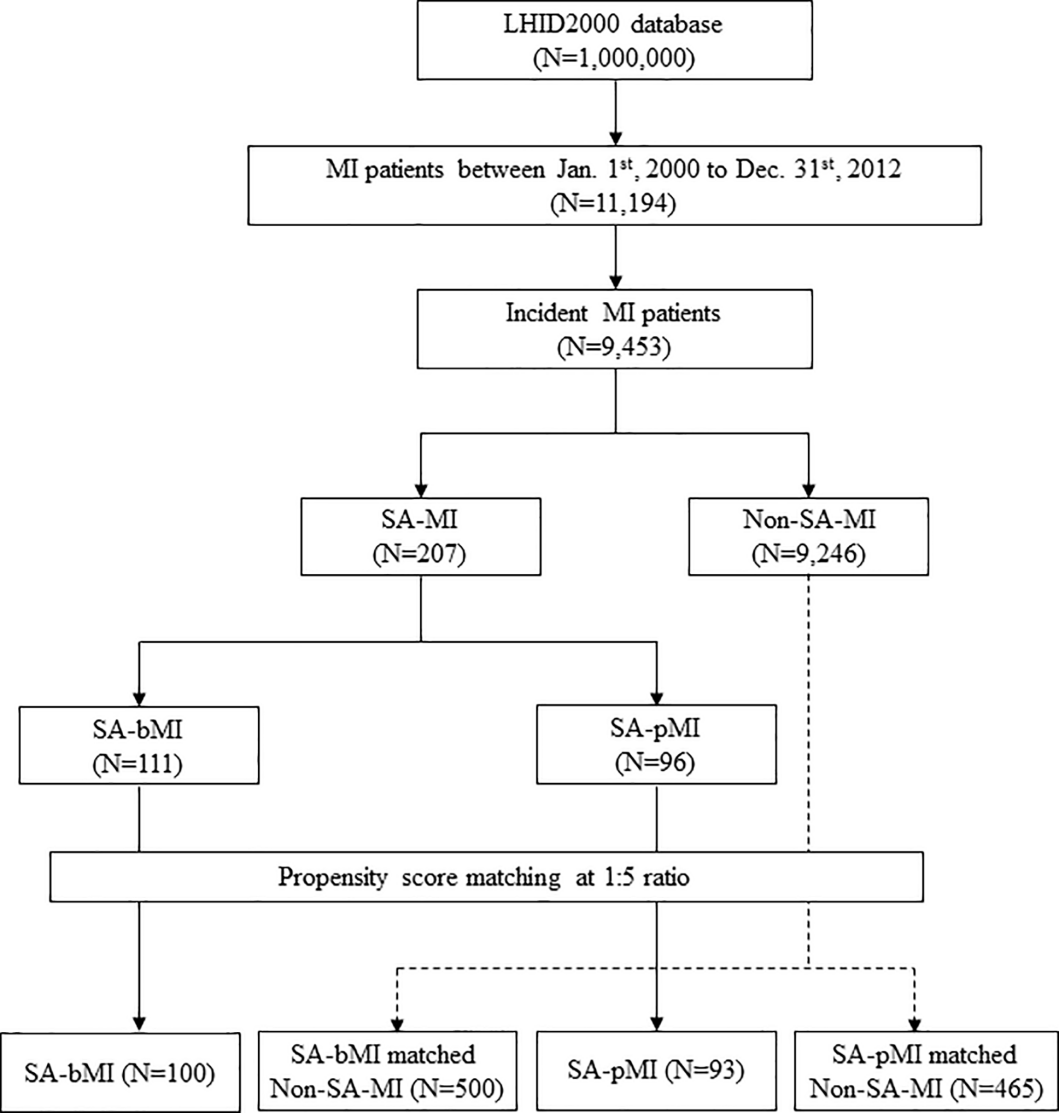
Flowchart of patient groups. LHID: Longitudinal Health Insurance Database, MI: myocardial infarction, SA: sleep apnea, PSG: polysomnography, SA-bMI: SA diagnosed before incident MI; SA-pMI: SA diagnosed post-incident MI.

The flowchart of patient groups is shown in Fig 1. Adult subjects (≧ 18 y/o) with incident MI between January 1^st^, 2000 and December 31^st^, 2012 were identified as the study cohort (MI cohort). Amongst the MI cohort, subjects with coexisting SA were identified as the study group (SA-MI), and subjects without SA (non-SA-MI) were identified as the control group. This study was approved by the Institutional Review Board of National Taiwan University Hospital (201309084RIND).

### Variables collection

Variables collected included gender, age, socioeconomic status, medical care utilization, comorbidity, concomitant medications, and treatment received (Table 1). Socioeconomic status was assessed by monthly income and urbanization level of residence.[19] Medical care utilization was assessed by times of outpatient visit or hospitalization one year before the index date. Comorbidity was identified by International Classification of Disease, Ninth Revision, Clinical Modification (ICD-9-CM) codes if a medical disease was diagnosed either once in inpatient claims or at least twice in outpatient claims one year before the index date, and was also presented as the Charlson Comorbidity Index (CCI).[20] Cardiovascular-related drug use after discharge from incident MI hospitalization was extracted, including aspirin, clopidogrel, warfarin, and statin. Intervention included PCI, coronary artery bypass graft surgery (CABG), and thrombolytic therapy.

**Table 1:** Baseline characteristics of the study groups.

Characteristics	SA-MI n = 207	Non-SA-MI n = 9,246	SA-bMI n = 111	SA-pMI n = 96
Male	165 (80)	6,196 (67)*	83 (75)	82 (85)
Age, y/o^#^	63±13	68±14*	65±14	63±12
Socioeconomic status				
Monthly income		*		
Dependent	10 (5)	1,108 (12)	5 (4)	5 (5)
NT$ <19,100	42 (20)	2,228 (24)	23 (21)	19 (20)
NT$ 19,100–42,000	97 (47)	4,850 (52)	50 (45)	47 (49)
NT$ >42,000	58 (28)	1,060 (12)	33 (30)	25 (26)
Urbanization level of residence		*		
1 (most urbanized)	165 (80)	6,577 (71)	88 (79)	77 (80)
2	32 (15)	1,766 (19)	17 (15)	15 (16)
3	8 (4)	588 (6)	4 (4)	4 (4)
4 (least urbanized)	2 (1)	315 (4)	2 (2)	0 (0)
Medical utilization				
Outpatient clinics, times^#^	33±24	27±21*	37±26	37±27
Hospitalization, times^#^	1.70±1.94	1.55±1.46	1.96±2.38	1.38±1.53^†^
Comorbidities				
Hypertension	147 (71)	5,524 (60)*	85 (77)	71 (74)
Diabetes	70 (34)	3,278 (35)	43 (39)	31 (33)
COPD	36 (17)	1,226 (13)	27 (24)	9 (9) ^†^
Asthma	17 (8)	568 (6)	10 (9)	9 (9)
Dysrhythmia	31 (15)	1,268 (14)	15 (14)	19 (20)
CVA	23 (11)	977 (11)	19 (17)	6 (6) ^†^
CKD	36 (17)	1,142 (12)*	21 (19)	18 (19)
Malignancy	13 (6)	564 (6)	10 (9)	3 (3)
Dyslipidemia	80 (39)	1,936 (21)*	47 (42)	54 (56) ^†^
CAD	161 (78)	6,419 (70)*	84 (76)	79 (82)
PAOD	4 (2)	148 (2)	2 (2)	2 (2)
Charlson comorbidity index score				^†^
0	0 (0)	0 (0)	0 (0)	8 (8)
1	41 (20)	1,950 (21)	17 (15)	26 (27)
2	49 (24)	2,062 (22)	19 (17)	17 (18)
≧3	117 (56)	5,234 (57)	75 (68)	45 (47)
Concomitant medications				
Aspirin	160 (78)	6,100 (66)*	87 (78)	73 (76)
Clopidogrel	82 (40)	2,515 (27)*	49 (44)	33 (34)
Warfarin	6 (3)	204 (2)	3 (3)	3 (3)
Statin	61 (29)	1,744 (19)	36 (33)	25 (26)
Treatment received				
PCI	94 (45)	3,661 (40)	49 (44)	45 (47)
CABG	15 (7)	475 (5)	7 (6)	8 (8)
Thrombolytic therapy	9 (4)	441 (5)	1 (1)	8 (8) ^†^
CPAP	64 (31)		30 (27)	34 (35)

Data are n (%) unless otherwise indicated.

^#^ Values presented as means ± standard deviation.

* p < 0.05. SA-MI comparing to non-SA-MI.

^†^ p < 0.05. SA-bMI comparing to SA-pMI.

Abbreviations: SA, sleep apnea; MI, myocardial infarction; SA-bMI, sleep apnea diagnosed before incident MI; SA-pMI, sleep apnea diagnosed post-incident MI; NT$, New Taiwan dollars; COPD, chronic obstructive pulmonary disease; CVA, cerebrovascular accident; CKD, chronic kidney disease; CAD, coronary artery disease; PAOD, peripheral arterial obstructive disease; PCI, Percutaneous coronary intervention; CABG, Coronary artery bypass graft surgery. CPAP, continuous positive airway pressure

### Identification of study and validation

The diagnosis and the corresponding ICD-9-CM codes are listed in S1 Table. The diagnosis of MI was identified by ICD-9-CM code 410, while the diagnosis of SA was identified by ICD-9-CM codes 780.51, 780.53, 780.57, 327.23.

The diagnosis of MI with ICD-9-CM coding was validated in 300 inpatients randomly selected between 2003 and 2012 from the claims database of the National Taiwan University Hospital (NTUH), a 2400-bed tertiary referral hospital. The contents of NTUH inpatient claims database were similar to those of NHIRD’s inpatient claims database. The diagnoses of MI were confirmed by reviewing clinical records according to consensus guidelines.[21] The diagnoses were confirmed in 96% of the patients, supporting the validity of ICD coding for the identification of MI (Cohen κ coefficient: 0.96, 95% CI: 0.94–0.98).

The diagnoses of SA were validated in 7,518 subjects of SA diagnosed with ICD-9-CM coding in NTUH from 2003 to 2012. For the 5,430 (72%) subjects who underwent in-lab polysomnography (PSG), 4,424 (81.5%) subjects had apnea-hypopnea index (AHI) values ≥ 5/hr, including 4,379 (99%) obstructive SA and 45 (1%) central SA, supporting the validity of ICD coding for the identification of SA. The diagnosis of SA has also been validated at the different institutions where the predictive value of 87–88% was reported [18, 22].

### Matching of comparison groups

To investigate the temporal effect of SA, MI subjects with coexistent SA were divided into two groups: SA diagnosed before incident MI (SA-bMI) and SA diagnosed post-incident MI (SA-pMI). The comparison groups were matched for each group separately at a ratio of five non-SA-MI subjects per SA-MI patient.[23] Propensity score matching (PSM) was performed based on age, gender, socioeconomic status, comorbidity, concomitant medications, and treatment using a multivariable logistic regression model. A nearest-neighbor-matching algorithm with a “greedy” heuristic was used to match the subjects. Matching occurs if the difference in the logits was within the caliper width of 0.2 standard deviation of the propensity scores. For comparing the SA-bMI and matched non-SA-MI groups, the date of incident MI was defined as the index date. For comparing the SA-pMI and the matched non-SA-MI groups, the date of SA diagnosis was defined as the index date, and the comparison group was selected from the non-SA-MI subjects who survived from the date of incident MI to the index date, in order to avoid immortal time bias. All of the enrolled subjects were followed from their index dates until death or Dec. 31^st^, 2012.

### Outcome

The endpoint of this study was all-cause mortality or major cardiac and cerebrovascular events (MACCEs), which was defined as composites of repeated MI (re-MI), coronary revascularization, hospitalization for ischemic heart disease (IHD), or stroke. Coronary revascularization was defined as repeated PCI or CABG. IHD was defined as the presence of sudden death, ventricular arrhythmia, angina, heart failure, or complications of ischemic cardiovascular disease, which includes rupture of chordae tendineae or papillary muscle, acquired septal defect, acquired mural thrombus, or carditis (S1 Table). Death was defined by the withdraw of the subject from the NHI program, and endpoints other than death were identified with ICD-9-CM codes.[24] The risk for morality and MACCEs was stratified by whether the subject received CPAP treatment or not where CPAP titration (NHI code 17008B and 57023B) was used as a surrogate maker for CPAP treatment.

### Statistical analysis

The chi-square (or Fisher’s exact test) and t-tests were used to determine the difference of categorical and continuous variables. The cumulative incidence curves were constructed using the Kaplan-Meier method and compared using the log-rank test. The Cox proportional hazards model with adjustment of unbalanced parameters was used to analyze the association of SA and the outcomes, and the results were presented as hazard ratio (HR) and 95% confidence interval (CI). Cubic spline functions were used to investigate the temporal effect on the prognosis of the SA-pMI group.[25] Based on cutting time point chosen from cubic spline curve, we stratified the SA-pMI group into two subgroups (early SA-pMI group and late SA-pMI group), and the outcome analysis was repeated in these two subgroups. All statistical analysis was conducted using SAS Version 9.3 (SAS Institute, Cary, NC) and a two-sided value of p < 0.05 was considered significant.

## Results

Between January 1^st^, 2000 and December 31^st^, 2012, 9,453 subjects were identified as the incident MI cohort, comprised of 207 subjects who were also diagnosed with SA (SA-MI) and 9,426 subjects who had never been diagnosed with SA (non-SA-MI) (Fig 1). The baseline characteristics of the incident MI cohorts are shown in Table 1. The follow-up duration was 56.2 ± 43.2 months for the SA-MI group and 40.8 ± 43.2 months for the non-SA-MI group. The prevalence of SA decreased in the MI subjects while it increased in the general population. The average prevalence of SA in MI was 2.2% over the 12-year study period while it was 1.19% (11,972 of 100,000 persons) in the general population (Figure A in S1 Fig). The overall percentage of SA-MI subjects undergoing PSG (1.5% or 139 of 9,453) during the 2000 to 2012 period was higher than that in the general population (0.8% or 8,331 of 1,000,000, Figure B in S1 Fig). However, considering the yearly increase in both MI prevalence and SA diagnosis in the general population, the ratio of the PSG percentage among MI subjects in reference to that in the general population declined year by year (Figure C in S1 Fig).

**Fig 2:**
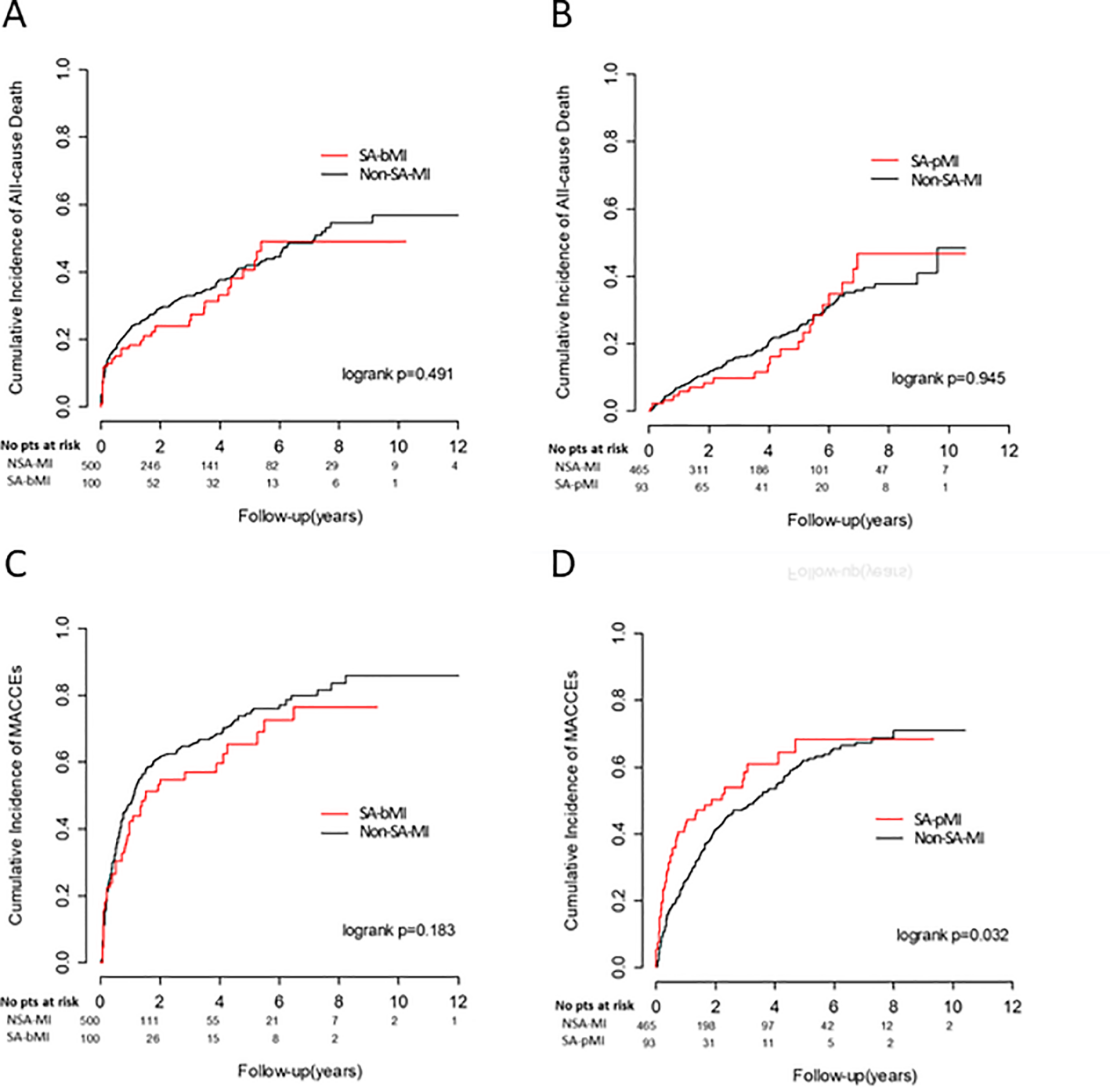
Cumulative incidence of all-cause death and major adverse cardiac and cerebrovascular events (MACCEs) amongst patients with sleep apnea diagnosed before incident myocardial infarction (SA-bMI) (A and C) or post myocardial infarction (SA-pMI) (B and D) with follow-up period starting from the index date.

The 207 SA-MI subjects were divided into two groups according to the timing of SA diagnosis in respect to MI: subjects with SA diagnosed before incident MI (SA-bMI, n = 111), and subjects with SA diagnosed post-incident MI (SA-pMI, n = 96). The average duration from MI to SA diagnosis was 3.71 years and 2.74 years in SA-bMI and SA-pMI groups, respectively. Comparing between these two groups, subjects of the SA-bMI group had higher medical utilization and higher CCI score, whereas a higher percentage of SA-pMI subjects received thrombolytic therapy during incident MI (Table 1). PSM was used to select matched control subjects from the non-SA-MI group. After PSM, 100 SA-bMI were matched to 500 non-SA-MI comparison subjects, and 93 SA-pMI were matched to 465 non-SA-MI subjects at baseline demographics, except a slightly higher proportion of SA-pMI subjects used clopidogrel compared with the matched comparison group (Table 2). The numbers of all-cause mortality and MACCEs for each group during the follow-up period are shown in Table 3, and the cumulative incidences of all-cause death and MACCEs are plotted in Fig 2. The risk of all-cause mortality was similar between the SA-bMI and SA-pMI groups and the respective comparison groups (Fig 2A and 2B). The risk for MACCEs significantly increased in the SA-pMI group (p = 0.032, Fig 2D), but not in SA-bMI (p = 0.183, Fig 2C).

**Table 2:** Baseline characteristics of patients with coexisting sleep apnea diagnosed before or post incident myocardial infarction and their propensity score–matched comparison groups.

Matching Parameters	SA-bMI n = 100	Comparison n = 500	p-value	SA-pMI n = 93	Comparison n = 465	p-value
Male	72 (72)	360 (72)	1.000	79 (85)	385 (83)	0.609
Age, y/o^#^	66±14	66±13	0.557	63±12	64±13	0.392
Socioeconomic status						
Monthly income			0.821			0.554
Dependent	5 (5)	27 (5)		5 (5)	24 (5)	
NT$ <19,100	23 (23)	109 (22)		17 (18)	94 (20)	
NT$ 19,100–42,000	49 (49)	227 (46)		46 (50)	240 (52)	
NT$ >42,000	23 (23)	137 (27)		25 (27)	107 (23)	
Urbanization level of residence			0.888			0.860
1 (most urbanized)	79 (79)	409 (82)		74 (80)	368 (79)	
2	15 (15)	67 (13)		15 (16)	74 (16)	
3	4 (4)	14 (3)		4 (4)	23 (5)	
4 (least urbanized)	2 (2)	10 (2)		0 (0)	0 (0)	
Medical utilization						
Outpatient clinics, times^#^	37±25	33±26	0.217	35±26	36±27	0.846
Hospitalization, times^#^	2.01±2.46	1.76±1.69	0.332	1.30±1.45	1.21±2.21	0.629
Comorbidities						
Hypertension	74 (74)	360 (72)	0.775	70 (75)	324 (70)	0.273
Diabetes	37 (37)	197 (39)	0.736	31 (33)	131 (28)	0.322
COPD	27 (27)	95 (19)	0.093	8 (9)	42 (9)	0.947
Asthma	9 (9)	33 (7)	0.520	7 (8)	46 (10)	0.466
Dysrhythmia	13 (13)	66 (13)	1.000	19 (20)	85 (18)	0.630
CVA	16 (16)	63 (13)	0.450	6 (6)	25 (5)	0.685
CKD	18 (18)	87 (17)	1.000	17 (18)	66 (14)	0.323
Malignancy	9 (9)	38 (8)	0.786	3 (3)	14 (3)	1.000
Dyslipidemia	37 (37)	210 (42)	0.414	52 (56)	246 (53)	0.595
CAD	73 (73)	383 (77)	0.521	77 (83)	362 (78)	0.278
PAOD	1 (1)	8 (2)	1.000	2 (2)	6 (1)	0.626
Charlson comorbidity index score			0.470			0.310
0	0 (0)	0 (0)		8 (9)	47 (10)	
1	14 (14)	96 (19)		25 (27)	149 (32)	
2	19 (19)	88 (18)		17 (18)	73 (16)	
≧3	67 (67)	316 (83)		43 (46)	196 (42)	
Concomitant medications						
Aspirin	76 (76)	391 (78)	0.725	71 (76)	351 (75)	0.860
Clopidogrel	41 (41)	246 (49)	0.165	32 (34)	109 (23)	0.031^†^
Warfarin	3 (3)	12 (2)	0.725	3 (3)	15 (3)	1.000
Statin	30 (30)	173 (35)	0.440	24 (26)	87 (19)	0.127
Treatment received						
PCI	44 (44)	249 (50)	0.342	45 (48)	199 (43)	0.322
CABG	5 (5)	28 (6)	1.000	7 (8)	36 (8)	0.943
Thrombolytic therapy	1 (1)	6 (1)	1.000	7 (8)	63 (14)	0.091
CPAP	26 (26)			33 (35)		

Data are n (%) unless otherwise indicated.

^#^ Values presented as means ± standard deviation.

^†^ p < 0.05. SA-bMI comparing to SA-pMI

Abbreviations: SA, sleep apnea; MI, myocardial infarction; SA-bMI, sleep apnea diagnosed before incident MI; SA-pMI, sleep apnea diagnosed post-incident MI; NT$, New Taiwan dollars; COPD, chronic obstructive pulmonary disease; CVA, cerebrovascular accident; CKD, chronic kidney disease; CAD, coronary artery disease; PAOD, peripheral arterial obstructive disease; PCI, Percutaneous coronary intervention; CABG, Coronary artery bypass graft surgery; CPAP: continuous positive airway pressure

**Table 3:** The number of all-cause mortality and the first-event of major adverse cardiac and cerebrovascular events in patients with coexisting sleep apnea diagnosed before or post incident myocardial infarction and their propensity score–matched comparisons.

Outcome parameters, n (%)	SA-bMI n = 100	Comparison n = 500	SA-pMI n = 93	Comparison n = 465
All-cause mortality	33 (33)	182 (36.4)	21 (22.6)	103 (22.2)
MACCEs	50 (50)	275 (55)	50 (53.8)	225 (48.4)
Re-MI or revascularization	30 (30)	154 (30.8)	31 (33.3)	98 (21.1)
IHD hospitalization	17 (17)	109 (21.8)	17 (18.3)	113 (24.3)
Stroke	3 (3)	12 (2.4)	2 (2.2)	14 (3)

Abbreviations: SA-bMI, sleep apnea diagnosed before incident myocardial infarction; SA-pMI, sleep apnea diagnosed post-incident myocardial infarction; MACCEs, major adverse cardiac and cerebrovascular events; re-MI, repeated myocardial infarction; IHD, ischemic heart disease.

**Fig 3:**
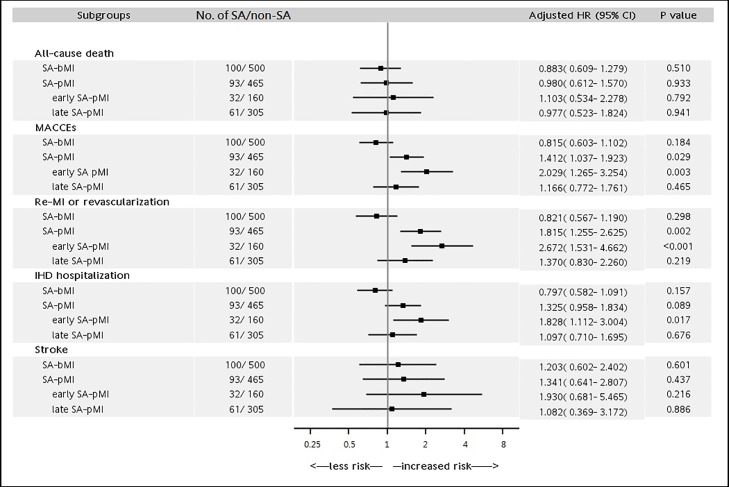
Risk of developing the all-cause death or major adverse cardiac and cerebrovascular events (MACCEs) stratified by the temporal sequence and the time interval between sleep apnea (SA) and incident myocardial infarction (MI). non-SA: non-sleep apnea; SA-bMI: SA diagnosed before incident MI; SA-pMI: SA diagnosed post-incident MI; early SA-pMI: SA diagnosed within one year after MI; late SA-pMI: SA diagnosed beyond one year after MI; IHD: ischemic heart disease. HR was calculated with adjustment for clopidogrel use in SA-pMI.

The adjusted HRs of all-cause mortality and MACCEs and the composites were analyzed with the Cox proportional hazards mode and listed in Fig 3. SA was not associated with increased risk of mortality or stroke in either the SA-bMI or SA-pMI group. SA-pMI was associated with increased risk of f MACCEs (HR: 1.412, 95% CI: 1.037~1.923, p = 0.029) and re-MI or revascularization (HR: 1.815, 95% CI: 1.255~2.625, p = 0.002), but SA-bMI was not.

The time-varying hazard of SA amongst MI subjects was estimated using the cubic spline function [25]. The cubic spline curve showed that subjects who were diagnosed with SA within one year after incident MI had the highest HR for MACCEs (S2 Fig). Subjects of SA-pMI were further stratified by a 1-year cutoff into the early SA-pMI group (n = 32) and the late SA-pMI group (n = 61). The average duration from MI to SA diagnosis was 4.54 months and 3.99 years in early and late SA-pMI groups, respectively. The early SA-pMI group showed increased risk for MACCE (HR: 2.029, 95% CI: 1.265~3.254, p = 0.003), including re-MI or revascularization (HR: 2.672, 95% CI: 1.531~4.662, p<0.001) and IHD hospitalization (HR: 1.828, 95% CI: 1.112~3.004, p = 0.017), but the late SA-pMI group did not (Fig 3).

In SA-bMI group, CPAP treatment was not associated with risk for MACCEs. On the contrary, in SA-pMI group, the risk of MACCEs, mainly for re-MI or revascularization, increased among SA patients who were not treated with CPAP (HR of MACCEs: 1.483, 95% CI: 1.007–2.186; HR of re-MI or revascularization: 2.220, 95% CI: 1.411–3.492) (Table 4).

**Table 4:** Risk of developing all-cause death or major adverse cardiac and cerebrovascular events stratified by with and without continuous positive airway pressure therapy among different temporal sequence between sleep apnea and incident myocardial infarction, compared with the corresponding propensity score–matched comparison group.

Adjusted HR	With CPAP therapy				Without CPAP therapy			
	SA-bMI	SA-pMI	Early SA-pMI	Late SA-pMI	SA-bMI	SA-pMI	Early SA-pMI	Late SA-pMI
	n = 26	n = 33	n = 11	n = 22	n = 74	n = 60	n = 21	n = 39
All-cause mortality	1.466 (0.714–3.009)	1.748 (0.881–3.470)	1.215 (0.371–3.981)	1.664 (0.697–3.969)	0.749 (0.483–1.163)	0.617 (0.318–1.198)	0.761 (0.296–1.956)	0.544 (0.214–1.382)
MACCEs	0.881 (0.486–1.598)	1.292 (0.775–2.155)	1.301 (0.543–3.116)	1.116 (0.571–2.182)	0.786 (0.553–1.117)	1.483 (1.007–2.186)*	2.259 (1.273–4.007)*	1.151 (0.682–1.942)
Re-MI or revascularization	0.690 (0.327–1.456)	1.432 (0.741–2.765)	1.193 (0.385–3.699)	1.357 (0.580–3.173)	0.866 (0.564–1.328)	2.220 (1.411–3.492)*	3.019 (1.582–5.760)*	1.457 (0.783–2.710)
IHD hospitalization	1.021 (0.559–1.864)	1.439 (0.858–2.411)	1.366 (0.572–3.264)	1.323 (0.674–2.598)	0.723 (0.500–1.048)	1.246 (0.819–1.895)	1.789 (0.973–3.290)	0.940 (0.530–1.666)
Stroke	1.251 (0.258–6.068)	0.907 (0.201–4.092)	5.104 (0.538–48.439)	-	1.136 (0.524–2.460)	1.520 (0.650–3.557)	1.443 (0.397–5.248)	1.651 (0.537–5.074)

Data was expressed as HR (95% CI)

* p < 0.05.

Abbreviations: HR, hazard ratio; CI, confidence interval; CPAP, continuous positive airway pressure; SA-bMI, sleep apnea diagnosed before incident myocardial infarction; SA-pMI, sleep apnea diagnosed post-incident myocardial infarction; early SA-pMI: SA diagnosed within one year after MI; late SA-pMI: SA diagnosed beyond one year after MI; MACCEs, major adverse cardiac and cerebrovascular events; re-MI, repeated myocardial infarction; IHD, ischemic heart disease

## Discussion

This study explored the temporal association between SA diagnosis, all-cause mortality, and first-event MACCEs after incident MI in a large, population-based, free from selection bias, cohort with the median follow-up period of 4.2 years. Our results showed that the SA diagnosis was not associated with all-cause mortality regardless of the timing of SA diagnosis. SA-pMI was associated with increased risk of MACCEs and its composites, including re-MI or revascularization and IHD hospitalization, especially when SA was diagnosed within one year after incident MI. Such an increased risk was not seen in SA-pMI treated with CPAP.

The prevalence of SA in MI has been reported to be up to 65.7%, depending on the severity of MI.[3] Even though the incidence of obstructive sleep apnea syndrome in Chinese has been reported to be 4.1% [26], the prevalence of SA in incident MI was only 2.2% in the present study. Such a low incidence is due to lack of awareness of SA among medical staffs, which was reflected by the low percentage of MI patient undergoing PSG (1.5%). Some subjects in our comparison group may also have undiagnosed SA, which would further underestimate the association between SA and risk of MACCEs.

Previous studies investigating the prevalence of SA in patients with MI or acute coronary syndrome have reported that obstructive apnea was the major type of sleep apnea.[4, 5] Central apnea accounted for less than 30% and was noted mostly within 2 weeks of MI.[27, 28] During a 6-month follow-up, obstructive apnea index did not change over time while central apnea index decreased over time.[5] In our early SA-pMI group, the mean duration between MI and SA diagnosis was 4.54 months so obstructive apnea should be the predominant type of apnea.

We showed that SA diagnosed prior to MI was not associated with increased risk of MACCEs, similar to the finding of a community-cohort based study reported by Gottlieb et al.[8] A couple of mechanisms contributed to the lack of association between SA and MACCEs after MI regardless of CPAP treatment. One is that SA related preconditioning may contribute to the development of collateral circulation. The other was that the diagnosis of SA may be associated with unrecognized behavioral modification which may dilute its negative impact on cardiovascular outcomes.

On the contrary, SA diagnosis post MI was associated with increased cardiovascular events, especially for those with SA diagnosed within one year after incident MI. Previous studies showed conflicting results for the impact of SA on outcomes in patients with MI or acute coronary syndrome (S2 Table) [10, 27, 29–37], mostly due to the heterogeneous study populations and inadequate follow-up periods. Our results were consistent with the findings of several studies that use AHI ≥ 15/hr to define obstructive SA and have long follow-up periods.[10, 29, 35–37] On the other hand, our results were different from those of studies using AHI or oxygen desaturation index (ODI) ≥ 5/hr as criteria for SA or assessing only short-term, in-hospital outcomes.[30–34] Most previous studies excluded patients in critical condition who need support of mechanical ventilation or oxygenation, with unstable hemodynamics, New York Heart Association (NYHA) Functional Classification 3–4, or high Killip III-IV. Our study contained no such selection bias and, with the median follow-up of 4.2 years, allowed a more comprehensive detection of SA-associated adverse cardiovascular events. And the population showing the highest risk in our study, the SA-pMI group, was similar to the SA subjects with pre-existing CAD that were included in a previous population-based study.[9] The study reported that SA caused a 1.6-fold increase in the risk of MACCEs, which was similar to our results (HR: 2.029 in Fig 3). Together, the findings support that SA could further compromise the insufficient coronary blood flow after MI and resulted in a worse cardiovascular outcome, which could be reversed by CPAP treatment.

The CPAP effect on MACCEs was inconclusive. A couple of randomized controlled trials showed CPAP treatment was not associated with risk of MACCEs except for subgroup who used CPAP ≥ 4hr/night.[38–42] The present study showed that the CPAP treatment was not associated with risk for MACCEs in SA-bMI which was comparable with two meta-analyses of RCTs.[41, 42] Our finding in SA-pMI that CPAP treatment was associated with lower risk for MACCEs was comparable with the findings in the observational studies.[43] It was not known if the reversal of risk in SA-pMI was related to the better compliance since the usage hour was not available in our database. Further prospective studies are needed to validate those findings.

This study is the first cohort study exploring the temporal association between SA diagnosis and the time interval between incident MI, and the mortality and cardiovascular event in a large population-based cohort with long-term follow-up. Such an approach avoided the selection bias common in single-institute, clinic-based studies or studies that recruited subjects with re-MI where the association of SA and MACCEs tended to be higher (S2 Table).[10, 27, 29–31, 34–37] Moreover, the follow-up period was adequate to allow ample time for the detection of possible adverse impacts of SA on the cardiovascular system.

There were several limitations in the present study. First, the ICD coding presented the timing of SA diagnosis instead of SA onset. Certain proportion of patients may have sleep disturbance prior to cardiovascular event without seeking medical help. That would underestimate the incidence of SA prior to incident MI and furthermore underestimate the association between SA-bMI and MACCEs. Second, the diagnosis of SA was solely relied on the ICD coded by physicians but not PSG in this study. Even though the validation showed good agreement between ICD coding and diagnosis of in-lab PSG, the severity of SA was not recorded in the database. Therefore, the association of SA severity and MACCEs could not be evaluated. Third, risk factors known to be associated with MI prognosis, including smoking, obesity, and family history of cardiovascular disease, were not available in the database. In our study, we adjusted comorbidities, such as chronic obstructive pulmonary disease, as a surrogate for cigarette smoking, in matching process because these diseases were highly associated with smoking. As for obesity, because of “obese paradox“, it would underestimate the harmful association of SA and MACCEs.[44] Nonetheless, the present study showed association between SA-pMI and MACCEs, especially with one year after incident MI, providing the basis for further investigation. A prospective, large-scale study is warranted to validate the aforementioned findings. Last, the database lacks the information of CPAP compliance. Therefore, it is not known if the difference in risk of MACCEs after CPAP treatment between SA-bMI and SA-pMI was related to compliance.

In conclusion, this community-based cohort study demonstrated that the temporal sequence and the time interval between SA diagnosis and incident MI was associated with the cardiovascular event after incident MI. SA diagnosed after MI, especially within one year, was associated with higher risk of MACCEs where CPAP treatment was associated with lower risk. Early assessment for the presence of SA after incident MI and early CPAP intervention may lower the risk of further adverse cardiovascular events.

## Supporting information

S1 Fig The trend of polysomnography (PSG) prescription and diagnosis of sleep apnea (SA) in total populations (black line) and patients with myocardial infarction (MI) (red line) from 2000 to 2012. A. Prevalence of MI and SA in MI patients and the general population. B. PSG prescription rate in MI patients and the general population. C. The ratio of PSG prescription percentage in MI patients in reference to that in the general population.
(PDF)

S2 Fig The interactions between sleep apnea (SA) diagnosis after incident MI and major adverse cardiac and cerebrovascular events (MACCEs) were analyzed by Cox proportional hazards regression models using cubic spline functions. Dash line: 95% confidence intervals. SA-pMI: SA diagnosed post incident myocardial infarction; Analysis included 96 SA-pMI and 9,246 non-SA-MI subjects.
(PDF)

S1 Table Diagnosis and corresponding International Classification of Diseases, Ninth Revision, Clinical Modification (ICD-9-CM) codes. (DOCX)

S2 Table Studies investigating impact of sleep apnea (SA) on cardiovascular prognosis after acute myocardial infarction (AMI) or acute coronary syndrome (ACS). (DOCX)

## Abbreviations

95% Cis: 95% confidence interval
AHI: apnea-hypopnea index
CABG: coronary artery bypass graft surgery
CAD: coronary artery disease
CCI: Charlson comorbidity index
CI: confidence interval
CIH: chronic intermittent hypoxia
CKD: chronic kidney disease
COPD: chronic obstructive pulmonary disease
CPAP: continuous positive airway pressure
CVA: cerebrovascular accident
HR: hazard ratio
ICD-9-CM: international classification of diseases, ninth revision, clinical modification
IHD: ischemic heart disease
LHID2000: Longitudinal Health Insurance Database 2000
MACCEs: major adverse cardiac and cerebrovascular events
MI: myocardial infarction
NHIRD: national health insurance research database
Non-SA: non-sleep apnea
NTUH: National Taiwan University Hospital
NT$: New Taiwan dollars
NYHA: New York Heart Association
ODI: oxygen desaturation index
PAOD: peripheral arterial obstructive disease
PCI: percutaneous coronary intervention
PSG: polysomnography
PSM: propensity score matching
Re-MI: repeated myocardial infarction
SA: sleep apnea
