# Supplementary figures and images for: Timely diagnosis and treatment of sleep apnea reduce cardiovascular sequelae in patients with myocardial infarction
Source: PLoS One. 2018 Jul 30;13(7):e0201493. doi: 10.1371/journal.pone.0201493 (PMC6066237; doi:10.1371/journal.pone.0201493)

A

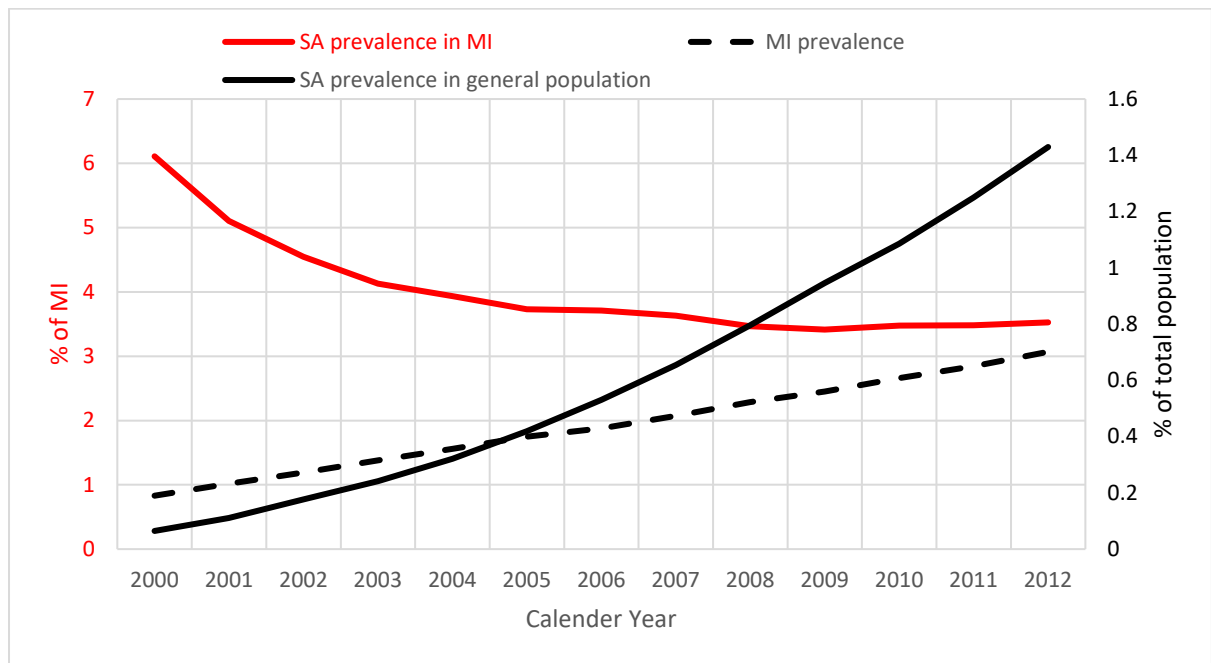

B

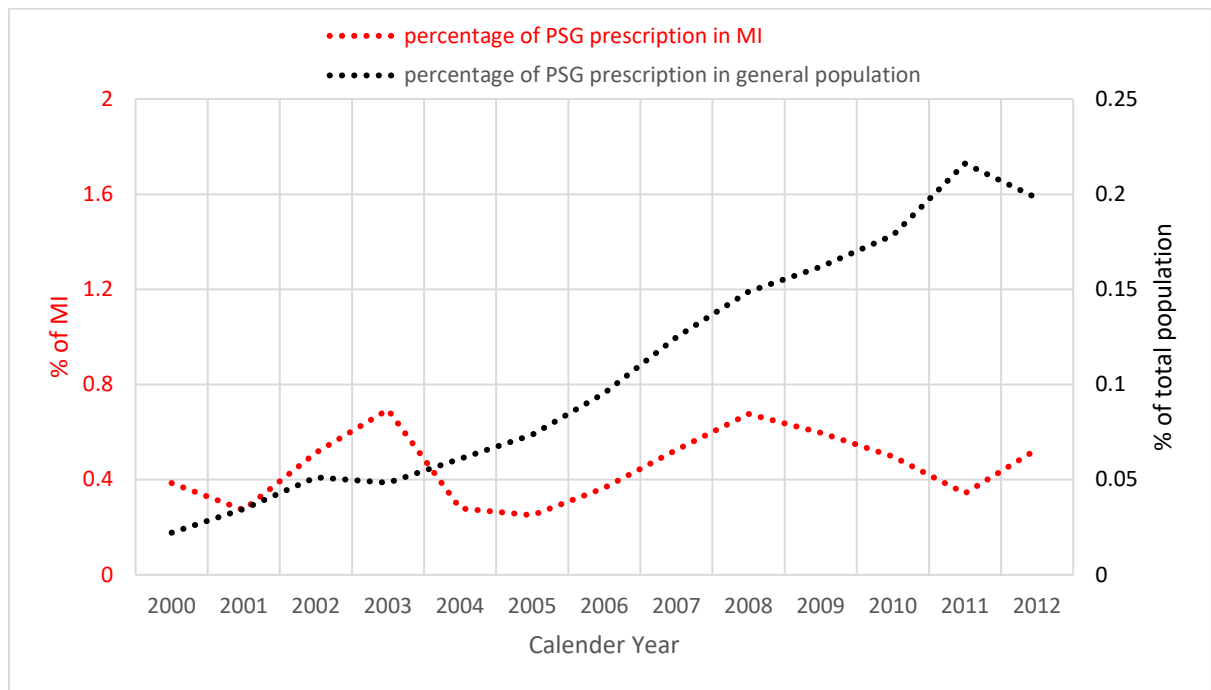

C

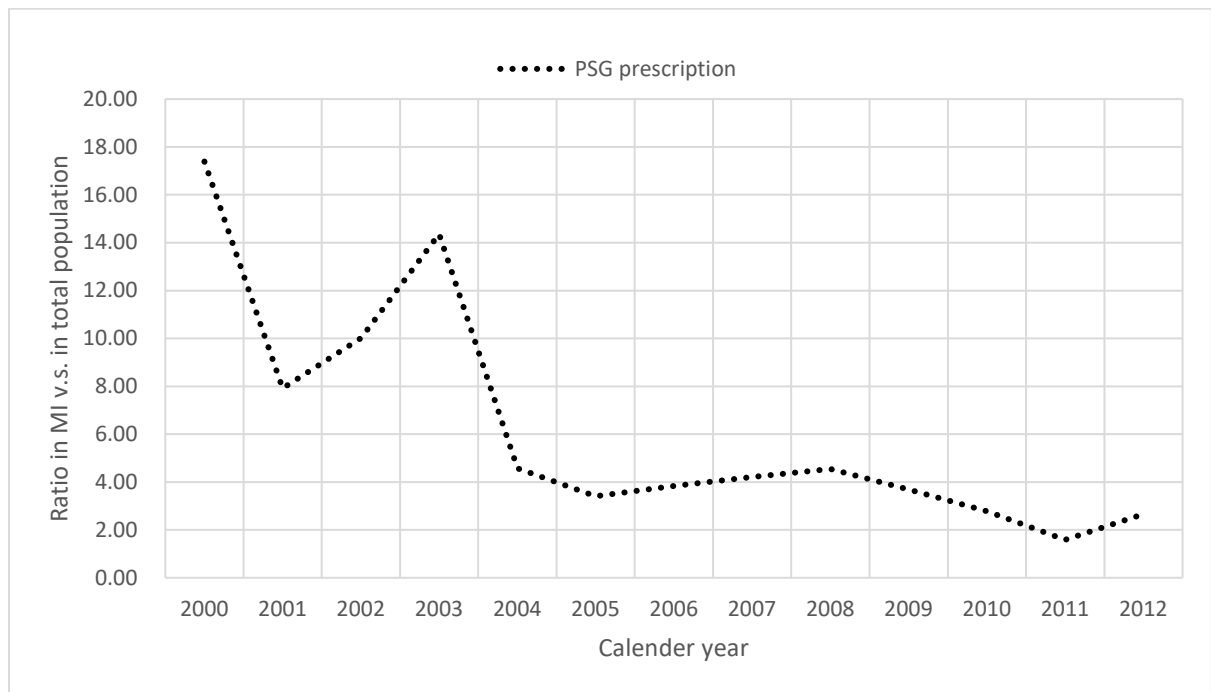

Supplement: S1 Fig — A. Prevalence of MI and SA in MI patients and the general population. B. PSG prescription rate in MI patients and the general population. C. The ratio of PSG prescription percentage in MI patients in reference to that in the general population. (PDF) [file pone.0201493.s001.pdf]

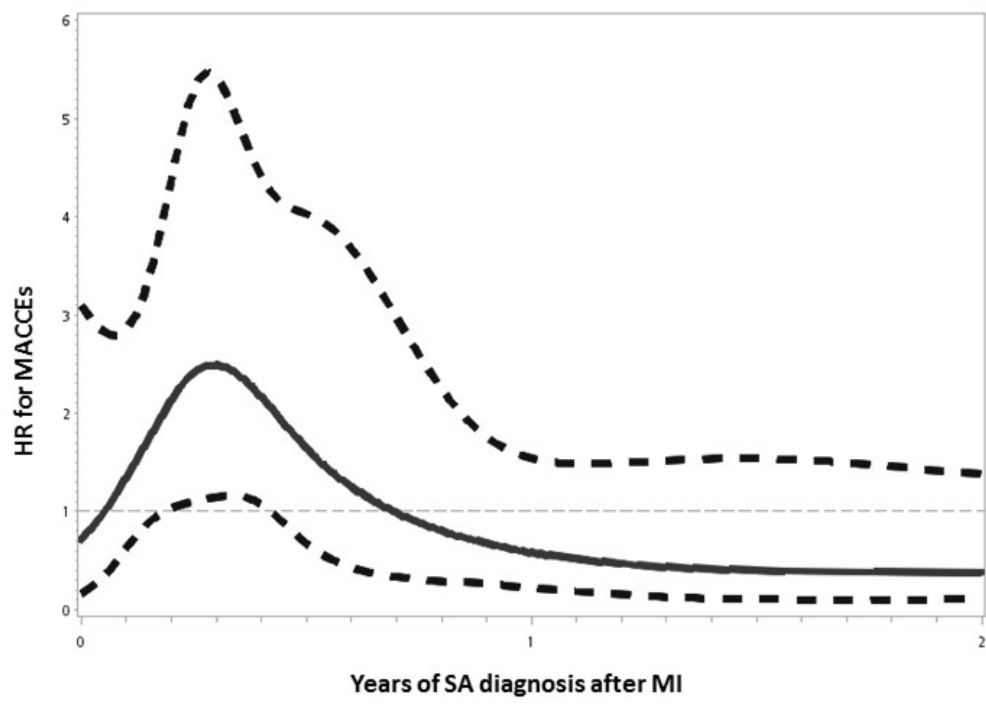

Supplement: S2 Fig — Dash line: 95% confidence intervals. SA-pMI: SA diagnosed post incident myocardial infarction; Analysis included 96 SA-pMI and 9,246 non-SA-MI subjects. (PDF) [file pone.0201493.s002.pdf]
