# Supplementary material for: Timely diagnosis and treatment of sleep apnea reduce cardiovascular sequelae in patients with myocardial infarction
Source: PLoS One. 2018 Jul 30;13(7):e0201493. doi: 10.1371/journal.pone.0201493 (PMC6066237; doi:10.1371/journal.pone.0201493)
Supplement: S1 Table — (DOCX) [file pone.0201493.s003.docx]

| **Diseases** | **ICD-9-CM codes** |
| --- | --- |
| Sleep apnea | 780.51, 780.53, 780.57, 327.23 |
| Myocardial infarction | 410 |
| Hypertension | 401-405 |
| Diabetic mellitus | 250 |
| Chronic obstructive pulmonary disease | 491, 492, 494, 496 |
| Asthma | 493 |
| Dysarrhythmia | 427 |
| Ischemic stroke | 433, 434, 436, 437.1 |
| Cerebrovascular disease | 430-438 |
| Chronic kidney disease | 581-583, 585, 586, 588 |
| Cancer | 140 -208 |
| Dyslipidemia | 272.0-272.5 |
| Coronary artery disease | 411-414 |
| Peripheral vascular disease | 443, 747.6 |
| Heart failure and pulmonary edema | 428, 518.4 |
| Shock | 458, 785.5 |
| Cardiac arrest | 427.5 |
| Dementia | 290 |
| Chronic pulmonary disease | 490-496, 500-505, 506.4 |
| Connective tissue diseases (Rheumatoid arthritis, systemic lupus erythematosus, systemic sclerosis, polymyositis) | 710.0, 710.1, 710.4, 714.0, 714.1, 714.2, 714.81, 725 |
| Peptic ulcer disease | 531-534 |
| Acquired immunodeficiency syndrome | 042 |
| Hemiplegia | 342, 344.1 |
| Liver diseases (chronic hepatitis or cirrhosis) |  |
| mild | 571.2, 571.4, 571.5, 571.6 |
| moderate-severe | 572.2-572.4, 572.8, 456.0, 456.1, 456.2 |
| Hospitalization due to ischemic heart disease |  |
| Angina | 413, 414, |
| Sudden cardiac death and ventricular arrhythmia | 427.4, 427.5, 798.1 798.2 |
| Heart failure | 428, 402.01, 402.11, 402.91, 429.9, 404.01, 404.03, 404.11, 404.13, 404.91, 404.93 |
| Cardiovascular disease | 429.2 |
| Complications (rupture of chordae tendineae or papillary muscle, acquired septal defect, acquired mural thrombus, carditis) | 429.5, 429.6, 429.7, 429.8 |
