# Supplementary material for: Timely diagnosis and treatment of sleep apnea reduce cardiovascular sequelae in patients with myocardial infarction
Source: PLoS One. 2018 Jul 30;13(7):e0201493. doi: 10.1371/journal.pone.0201493 (PMC6066237; doi:10.1371/journal.pone.0201493)
Supplement: S2 Table — (DOCX) [file pone.0201493.s004.docx]

| **Studies** | **Study cohort** | **No of SA/NSA pts** | **SA criteria** | **Duration of follow-up (months)** | **Including previous AMI history** | **Excluding critical cases^*^** | **Excluding previous SA history** | **Duration of sleep study from AMI** | **Results** |
| --- | --- | --- | --- | --- | --- | --- | --- | --- | --- |
| Marin JMl. 1998[1] | AMI | 55/196 | DI>=10  +EDS+heavy snoring | 1 (in hospital) | Yes | Yes | No | Within 2 months | No difference in incidence of major AMI complications (38.2% vs 34.2%, p>0.05) or in mortality (14.5% vs 12.2%, p>0.05) between SA and NSA |
| Mehra R. 2006[2] | ACS | 69/35 | AHI>=10 | 6 | Yes | Yes | Yes | Within 3 days | No significant difference in readmission and ER visits between SA and NSA: adjusted OR:1.08 (95%CI:0.27-4.26, p=0.92) |
| Yumino D. 2007[3] | ACS s/p PCI | 51/38 | AHI>=10 | 8.1 | Yes | Yes | No | 7-14 days | Higher incidence of MACEs (23.5% vs 5.3%, p=0.022) in SA: adjusted HR: 11.61 (95%CI:2.17-62.24) |
| Meng S. 2009[4] | ACS s/p PCI | 76/47 | AHI>=5 | 12 | No | Yes | No | Within 7 days | No significant difference in mortality.  No significant difference in incidence of MACCEs between SA/NSA (17.3% vs 10.4%) |
| Jesus EVS. 2010[5] | ACS | 94/106 | Berlin Q | in hospital | Yes | Yes | No | No sleep study | No significant difference in incidence of mortality (4.25% vs 0.94%, p=0.189), but higher risk of MACCEs in SA (18.08% vs 6.6%, p=0.016): adjusted OR: 2.657 (95%CI: 1.216-10.996, p=0.021) |
| Lee CH. 2011[6] | AMI s/p PCI | 44/61 | AHI>=30 | 18 | Yes | Yes | Yes | 2-5 days | One cardiac death only in SA.  Higher incidence of MACEs (15.9% vs 3.3%) in SA: adjusted HR: 5.36 (95%CI: 1.01-28.53, p=0.049) |
| Correia LCL. 2012[7] | ACS | 123/45 | Berlin Q | 0.3 (in hospital) | No | Yes | No | No sleep study | Higher in-hospital mortality in high SA probability (0% vs 7.2% vs 15%, p=0.008)  Higher incidence of MACEs in SA (18%, p=0.002): adjusted OR: 3.4 (95%CI:1.3-9, p=0.015) |
| Aronson D. 2014[8] | AMI | 116/64 | ODI>=5 | 68 | Yes | Yes | No | Within 5 days | No difference in MACEs and mortality between SA and NSA. |
| Loo G. 2014[9] | ACS s/p PCI | 24/44 | AHI>=15 | 24 | Yes | Yes | Yes | Within 30 days (Median 14 days) | No cardiac death.  Higher incidence of MACCEs in SA (34.9% vs 5.1%, p=0.008): adjusted HR:6.95 (95%CI:1.17-41.4, p=0.033) |
| Nakashima H. 2015[10] | AMI s/p PCI | 124/148 | AHI>=15 | 50.4 | No | Yes | No | 14-21 days | No difference in total and cardiac death.  Higher incidence of recurrent ACS (16% vs 7%, p=0.014) and MACEs (22% vs 11%, p=0.014) in SA: adjusted HR for recurrent ACS: 2.3 (95%CI:1.04-5.13, p=0.04), adjusted HR for MACEs: 1.82 (95%CI:0.96-3.46, p=0.068) |
| Leao S. 2016[11] | ACS | 46/27 | AHI>=15 | 75 | No | No | Yes | Median 55 days | 6 death in moderate to severe SA vs 3 death in NSA  Severe SA had higher incidence of MACEs (HR:3.58, 95%CI: 1.09-17.73) |

^*^ Critical case defined by patients needing support of mechanical ventilator or oxygenation, with unstable hemodynamics or NYHA class 3-4 or high Killip (3-4), or who could not answer questionnaire or stand up or discharge alive.

Abbreviations: PCI, percutaneous coronary intervention; DI, desaturation index; AHI, apnea-hypopnea index; ODI, oxygen desaturation index; EDS, excessive daytime sleepiness; SA, sleep apnea; NSA, non-sleep apnea; Cox PH, Cox proportional hazards; HR, hazard ratio; OR, odds ratio; MAC(C)Es, major adverse cardiac (and cerebrovascular) events.

**Reference of Supplements**

1. Marin JM, Carrizo SJ, Kogan I. Obstructive sleep apnea and acute myocardial infarction: clinical implications of the association. Sleep. 1998;21(8):809-15.

2. Mehra R, Principe-Rodriguez K, Kirchner HL, Strohl KP. Sleep apnea in acute coronary syndrome: high prevalence but low impact on 6-month outcome. Sleep Med. 2006;7(6):521-8.

3. Yumino D, Tsurumi Y, Takagi A, Suzuki K, Kasanuki H. Impact of obstructive sleep apnea on clinical and angiographic outcomes following percutaneous coronary intervention in patients with acute coronary syndrome. Am J Cardiol. 2007;99(1):26-30.

4. Meng S, Fang L, Wang CQ, Wang LS, Chen MT, Huang XH. Impact of obstructive sleep apnoea on clinical characteristics and outcomes in patients with acute coronary syndrome following percutaneous coronary intervention. J Int Med Res. 2009;37(5):1343-53.

5. Jesus EV, Dias-Filho EB, Mota Bde M, Souza L, Marques-Santos C, Rocha JB, et al. Suspicion of obstructive sleep apnea by Berlin Questionnaire predicts events in patients with acute coronary syndrome. Arq Bras Cardiol. 2010;95(3):313-20.

6. Lee CH, Khoo SM, Chan MY, Wong HB, Low AF, Phua QH, et al. Severe obstructive sleep apnea and outcomes following myocardial infarction. J Clin Sleep Med. 2011;7(6):616-21.

7. Correia LC, Souza AC, Garcia G, Sabino M, Brito M, Maraux M, et al. Obstructive sleep apnea affects hospital outcomes of patients with non-ST-elevation acute coronary syndromes. Sleep. 2012;35(9):1241-5A.

8. Aronson D, Nakhleh M, Zeidan-Shwiri T, Mutlak M, Lavie P, Lavie L. Clinical implications of sleep disordered breathing in acute myocardial infarction. PloS One. 2014;9(2):e88878.

9. Loo G, Tan AY, Koo CY, Tai BC, Richards M, Lee CH. Prognostic implication of obstructive sleep apnea diagnosed by post-discharge sleep study in patients presenting with acute coronary syndrome. Sleep Med. 2014;15(6):631-6.

10. Nakashima H, Kurobe M, Minami K, Furudono S, Uchida Y, Amenomori K, et al. Effects of moderate-to-severe obstructive sleep apnea on the clinical manifestations of plaque vulnerability and the progression of coronary atherosclerosis in patients with acute coronary syndrome. Eur Heart J Acute Cardiovasc Care. 2015;4(1):75-84.

11. Leao S, Conde B, Fontes P, Calvo T, Afonso A, Moreira I. Effect of Obstructive Sleep Apnea in Acute Coronary Syndrome. Am J Cardiol. 2016;117(7):1084-7.
